# First cases of European bat lyssavirus type 1 in Iberian serotine bats: Implications for the molecular epidemiology of bat rabies in Europe

**DOI:** 10.1371/journal.pntd.0006290

**Published:** 2018-04-23

**Authors:** Patricia Mingo-Casas, Virginia Sandonís, Elena Obón, José M. Berciano, Sonia Vázquez-Morón, Javier Juste, Juan E. Echevarría

**Affiliations:** 1 National Center for Microbiology, Instituto de Salud Carlos III, Majadahonda, Madrid, Spain; 2 Centre de Fauna Salvatge de Torreferrussa, Catalan Wildlife Service, Direcció General de Medi Natural-Forestal Catalana, Santa Perpètua de la Mogoda, Barcelona, Catalonia, Spain; 3 Estación Biológica de Doñana, CSIC, Sevilla, Andalusia, Spain; 4 CIBER de Epidemiología y Salud Pública, CIBERESP, Madrid, Spain; Instiute of Parasitology, University of Veterinary Medicine Vienna, AUSTRIA

## Abstract

Previous studies have shown that EBLV-1 strains exclusively hosted by *Eptesicus isabellinus* bats in the Iberian Peninsula cluster in a specific monophyletic group that is related to the EBLV-1b lineage found in the rest of Europe. More recently, enhanced passive surveillance has allowed the detection of the first EBLV-1 strains associated to *Eptesicus serotinus* south of the Pyrenees. The aim of this study is the reconstruction of the EBLV-1 phylogeny and phylodynamics in the Iberian Peninsula in the context of the European continent. We have sequenced 23 EBLV-1 strains detected on nine *E*. *serotinus* and 14 *E*. *isabellinus*. Phylogenetic analyses were performed on the first 400-bp-5’ fragment of the Nucleoprotein (N) gene together with other 162 sequences from Europe. Besides, fragments of the variable region of the phosphoprotein (P) gene and the glycoprotein-polymerase (G-L) intergenic region were studied on Spanish samples. Phylogenies show that two of the new EBLV-1a strains from Iberian *E*. *serotinus* clustered together with French strains from the North of the Pyrenees, suggesting a recent expansion southwards of this subtype. The remaining seven Iberian strains from *E*. *serotinus* grouped, instead, within the cluster linked, so far, to *E*. *isabellinus*, indicating that spatial distribution prevails over species specificity in explaining rabies distribution and supporting interspecific transmission. The structure found within the Iberian Peninsula for EBLV-1b is in concordance with that described previously for *E*. *isabellinus*. Finally, we have found that the current EBLV-1 European strains could have emerged only 175 years ago according to our evolutionary dynamics analyses.

## Author summary

Rabies is caused by at least fourteen different viruses of the genus Lyssavirus. Although the classical rabies virus transmitted by the dog accounts for most human cases, most lyssaviruses are hosted by bats, which are able to transmit the disease to humans. The European bat lyssaviruses 1 (EBLV-1) and 2 (EBLV-2) have caused human rabies in Europe, while more than 90% of the bat rabies cases have been reported from serotine bats (*Eptesicus serotinus*) infected by EBLV-1. These cases clearly accumulate in certain areas of Europe, but the factors driving this distribution are unknown. The evolutionary relationships among these viral strains provide valuable information, however, they are only partially known. The south of the Iberian Peninsula is the only location within Europe with an additional reservoir for EBLV-1, the isabelline serotine bat (*Eptesicus isabellinus*) which is present also in North Africa. In this study we have reconstructed the EBLV-1 phylogeny and phylodynamics in the Iberian Peninsula in the context of the European continent. Our results suggest that some lineages have longer evolutionary history in their distribution areas, than others which seem to be in the process of a geographical expansion.

## Introduction

Rabies is caused by viruses of the genus *Lyssavirus*, which includes so far fourteen species recognized by the International Committee on Taxonomy of Viruses (ICTV), plus the recently proposed *Lleida bat Lyssavirus* (LLEBV) [1] and Gannoruwa bat lyssavirus (GBLV) [2]. Three of the viruses of the phylogroup 1 are associated with European bats of the family *Vespertilionidae*: the *European bat Lyssaviruses 1* and *2* (EBLV-1 and EBLV-2) and the *Bokeloh bat Lyssavirus* (BBLV). Besides, the other two lyssaviruses described so far in Europe (*West Caucasian bat virus*, WCBV; and LLEBV) are hosted by the cave bat *Miniopterus schreibersii* (Family *Miniopteridae*) [1, 3]. Out of all of them, only EBLV-1 and EBLV-2 have caused rabies in humans, while more than 90% of the bat rabies cases have been reported from serotine bats (*E*. *serotinus*) infected by EBLV-1 [4]. EBLV-1 is divided in two main subtypes: EBLV-1a and EBLV-1b. The first one is found in an East-west axis from Ukraine to the north of France, while EBLV-1b is reported from France, southern Germany and the Netherlands [5]. EBLV-1a has been recently described also from Southern France [6]. Nevertheless, the evolutionary relationships among these viral subtypes in Western Europe are only partially known and previous attempts of evolutionary analyses [7] were hampered either by uneven available sampling or the dearth of molecular markers.

Two cryptic species of serotine bats (*E*. *serotinus* and *E*. *isabellinus*) have been lately found within the genus *Eptesicus* in the Iberian Peninsula [8]. While *E*. *serotinus* is distributed across Northern Iberia as well as the rest of Western Europe, the sibling species.

*E*. *isabellinus* is restricted to the Southern half of the Iberian Peninsula and Northern Africa [9]. So far, EBLV-1 infection has been declared only from *E*. *isabellinus* in Spain [7], forming an exclusive monophyletic clade, possibly related to the EBLV-1b subtype.

In this study, we described the first EBLV-1 associated to *E*. *serotinus* South of the Pyrenees, and show how the inclusion of these new strains in phylogenetic and phylodynamic analyses together with the use of newly developed molecular markers have substantially improved our present knowledge of EBLV-1 molecular epidemiology in Europe and the Iberian Peninsula.

## Materials and methods

### Ethics statement

No live animals were used for this study. All the work has been done with bat carcasses from, either animals directly submitted to the Laboratory by public health services, or admitted in wildlife care Centers, which submitted to the laboratory those bats which could not be recovered after treatment. Consequently, the need for approval by an IACUC/ethics committee does not apply.

### Samples

We have sequenced three fragments of the viral genome of 23 EBLV-1 strains detected in brains of nine *E*. *serotinus* and 14 *E*. *isabellinus* sent to the National Center for Microbiology (Majadahonda, Spain) for rabies diagnosis and that were found positive for *Lyssavirus* antigen by the fluorescence antibody test and real time polymerase chain reaction (RT-PCR) [10].

Morphological bat identification was confirmed in most cases by genomic sequencing of several diagnostic mtDNA fragments [9]: 69R99 (MG211681), 80R99 (MG211682), 56R00 (MG211683), 69R00Eis (MG211684), 211R07 (MG211685), 86R08 (MG211686), 2011Riglos (MG211687), 201127004 (MG211688), 201149008 (MG211689), 201238163 (MG211690), 201325895 (MG211691), 200928458 (MG211692), 201544034 (MG211693), 201548093 (MG211694), 201539228 (MG211695), 201427094 (MG211696), 201539226 (MG211697), 44R02 (MG211698), 292R07 (MG211699).

### Lyssavirus RT-PCR amplification methods

The 400-bp-5' terminal sequence of the nucleoprotein (N-400) gene was amplified and sequenced as described previously [7]. Besides, a 686-bp variable region of the phosphoprotein (P) and a 763-bp fragment including the glycoprotein-polymerase (G-L) intergenic region were amplified by EBLV-1 specific primers in nested RT-PCR reactions specific for each region (Table 1).

**Table 1:** Primers.

Primeras	Region	Sequence (5’ → 3’)	Position RV2416	Size
**1617 P_1F**	P	TGG AGG ATA GTC AAG CCC AC	1617–1636	769 pb
**2386 P_1R**	P	TAT CTG TTK ARA TCA TCTY GC	2386–2406	
**1671 P_2F**	P	CTG AGG ATA TTA AGA GGC TCA	1671–1691	686 pb
**2357 P_2R**	P	GCC YAR TTT CGC CGA ATT GAC	2357–2377	
**4633 GL_1F**	G-L	TCA CCT TCC AGA CAC CCA	4633–4652	896 pb
**5502 GL_1R**	G-L	TCA GGT CTG CTT CTG GCT CA	5502–5521	
**4712 GL_2F**	G-L	ATA TCT GTG CTT GCC CTT CT	4712–4731	763 pb
**5475 GL_2R**	G-L	CCA CCG GAT CAT CGT AAA CC	5465–5484	

### Phylogenetic analyses

All 23 N-400 sequences from Spain were aligned using the MAFFT software (http://mafft.cbrc.jp/alignment/software/) together with 162 sequences from other European countries available on GenBank (S1 Table). The fittest nucleotide substitution model was selected according to the Bayesian information criterion (BIC) using jModelTest v.2.1.10 (http://darwin.uvigo.es/our-software/). For the Iberian samples we selected the substitution models HKY, K80 + I, K80 for the alignments of the P, G-L and N400 markers respectively. Besides, a GTR model was selected for the alignment of all Iberian and European N400 fragments.

Only for the Iberian samples, the N, P and G-L fragments were concatenated into a 1809 base pair (bp) alignment using Sea View v.4.6. (Table 1) and a HKY nucleotide substitution model was selected from this concatenated alignment using Partition Finder v.1.1.1. (http://www.robertlanfear.com/partitionfinder/).

Phylogenetic relationships were reconstructed for each alignment using a Bayesian approach with the program MrBayes (version 3.1.2) with two runs of 1 x 10^6^ generations. Additional phylogenies were reconstructed using other three optimality criteria: Minimum Evolution (ME), Maximum Likelihood (ML) and Maximum Parsimony (MP). ME was obtained through a Neighbor-Joining algorithm with MEGA7 (http://www.megasoftware.net/), ML was constructed with PHYML software (http://www.atgc-montpellier.fr/phyml/) and finally, MP trees were inferred with PAUP* 4.0b10 (http://paup.csit.fsu.edu/) weighting transversions differentially according to the transitions/transversion ratio of the selected substitution model. Confidence in the topologies was inferred after 1,000 bootstrap replicates. Trees were edited using the FigTree v1.4.1 program (http://tree.bio.ed.ac.uk/software/figtree/).

Viral genetic geographic structure across Europe was examined for each lineage data set, EBLV-1a and EBLV-1b, through parsimony-based haplotype networks employing the Median Joining (MJ) algorithm implemented in Network (v5.0, Fluxus Technology (http://www.fluxus-engineering.com/sharenet.htm), without star-contraction pre-processing or post-processing clean-up options.

To reconstruct phylodynamic patterns of EBLV-1 we analysed the N-400 sequence dataset in a Bayesian framework implemented in the BEAST software (Bayesian Evolutionary Analysis Sampling Trees (http://beast.bio.ed.ac.uk/) [11]. For model parameters we follow Hughes 2008’s analyses that use similar sequences of the same marker and virus. Accordingly, evolution rates were assumed to fit an uncorrelated lognormal molecular clock [12] and two MCMC chains were run for 5 x 10^7^ generations each with trees and parameters sampled every 1000 steps. We used the SRD06 option for codon partition and substitution model according to the recommendation of Hughes 2008. This option includes an HKY substitution model and four gamma rates with two codon partitions for the alignment: first and second codons were analysed together and separately from the third codon. Analyses started with a random starting tree with a constant population size prior, according to Hughes 2008. Then, chains were run to an effective sample size (ESS) of parameters higher than 200 (following BEAST’s recommendations) and chains convergence was assessed from standard deviation and likelihood values using Tracer (http://tree.bio.ed.ac.uk/software/tracer/) with the first 10% of the trees discarded as burning. Bayesian credible intervals or 95% Highest Posterior Density (95% HDP) were inferred as uncertainty evaluation. Finally, the consensus phylogenetic tree was built with TreeAnnotator (BEAST software package) and edited in FigTree (http://tree.bio.ed.ac.uk/software/figtree/).

## Results and discussion

**Fig 1:**
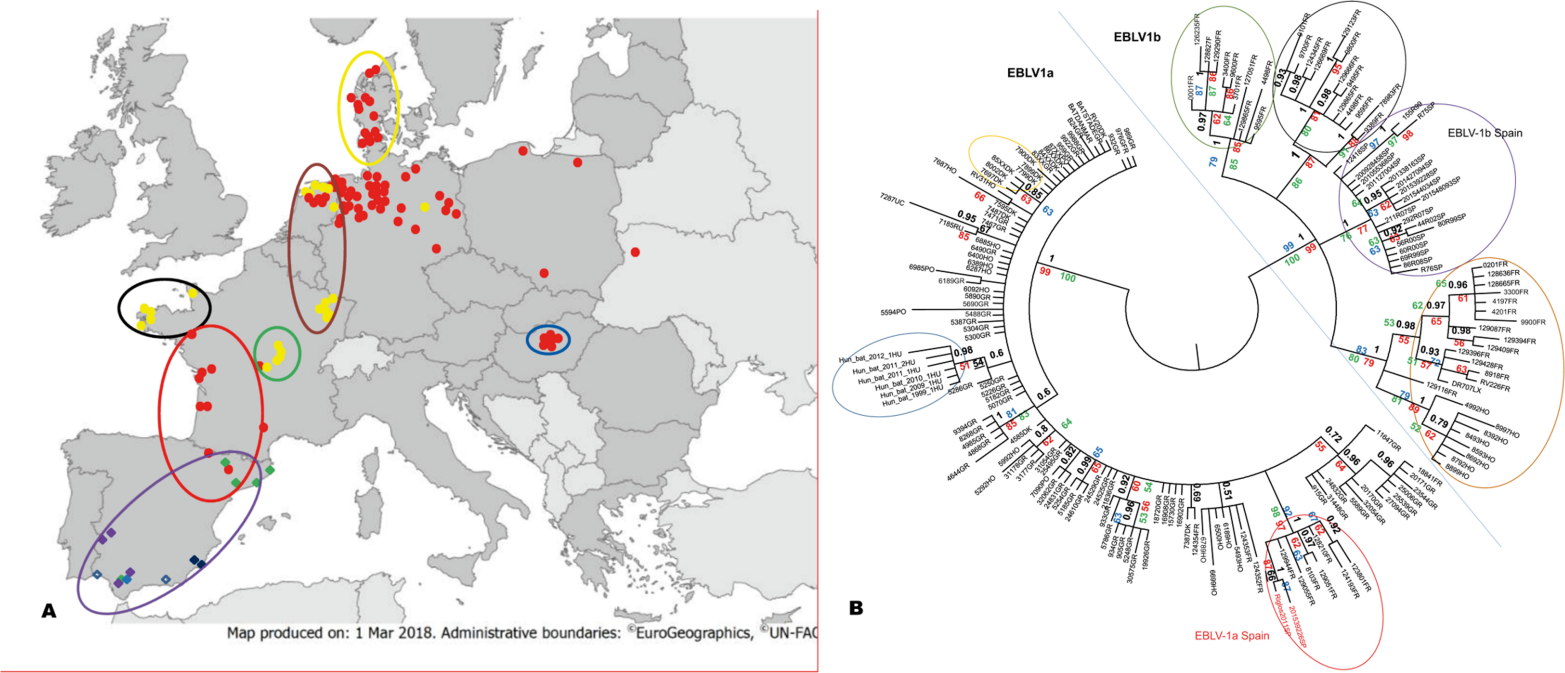
EBLV-1 in Europe. **1A:** Geographical distribution of the lineages described within the EBLV-1 rabies virus across Western Europe: EBLV-1a (red dots) along Northern, Central Europe. EBLV-1b (yellow dots) along Central Europe and the Iberian EBLV-1b, with a further subdivision between Southwestern major (purple diamonds), Southwestern minor (blue diamonds) Southeastern (black diamonds) and Northwestern. (green diamonds) strains. Two strains from the same location (R76, 292R07) not grouping in any of the previous lineages are represented by a dotted diamond. Colored circles correspond to the main groups identified in the phylogenetic reconstruction shown in Fig 1B. The base layer has been taken from https://mapmaker.ecdc.europa.eu/#, map produced on: 1 Mar 2018. Administrative boundaries: Eurogeographics, UN-FAO. **1B:** Midpoint-rooted Bayesian phylogenetic consensus reconstruction of European EBLV-1 based on a 400-bp-5' first fragment of the Nucleoprotein gene. Tree nodes show posterior probability (black) and also Maximum-Parsimony bootstrap support (red), Neighbor-Joining bootstrap support (blue) and Maximum-Likelihood bootstrap support (green). Colored circles correspond to the main phylogenetic groups.

**Fig 2:**
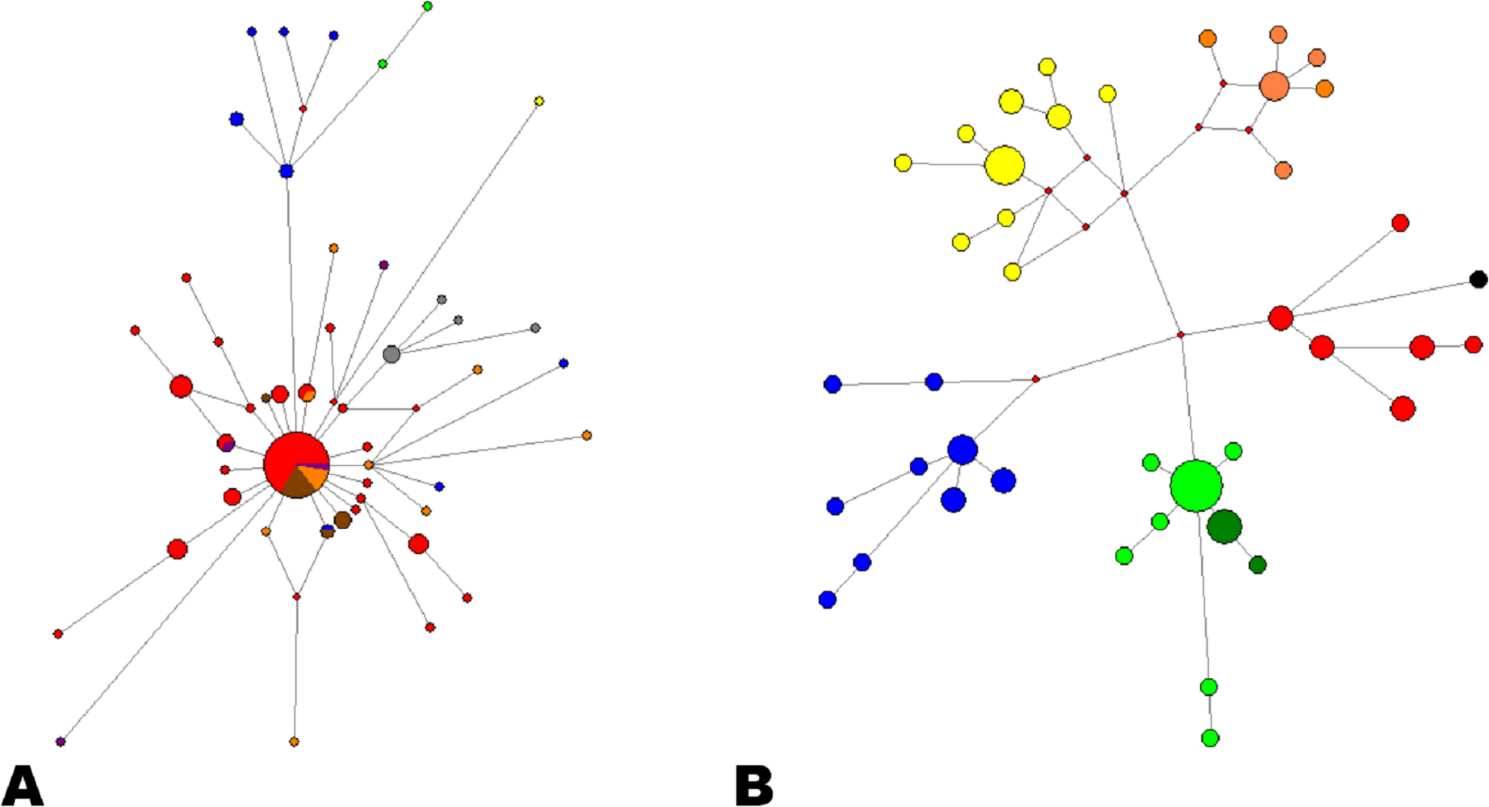
Viral genetic geographic structure across Europe through median-joining haplotype network. **2A:** EBLV-1a lineage. Circle’s sizes are proportional to the number of individuals sharing this particular haplotype and color code the regions of origin of haplotypes, Red: Germany; Dark Blue: France; Brown: Denmark; Grey: Hungary; Purple: Poland; Orange: The Netherlands; Light Green: Spain. Reconstructed haplotypes are represented as small red squares. **2B:** EBLV-1b lineage. Circle’s sizes are proportional to the number of individuals sharing this particular haplotype and colors code the regions of origin of haplotypes, Red: Central France; Dark Blue: North-west France; Yellow: North-east France; Black: Germany; Orange: The Netherlands; Light Green: Southern Spain; Dark Green: Northern Spain. Reconstructed haplotypes are represented as small red squares.

EBLV-1 is mostly found associated with the bat *E*. *serotinus* north of the high mountain ranges of Southern Europe, such as the Alps or the Pyrenees, which seem to hinder virus expansion to the south. However, a group of sequences from Southern Spain were found to cluster in a monophyletic group associated with a different bat, the sibling species *E*. *isabellinus* [7]. In this study, we report for the first time nine EBLV-1 strains found in *E*. *serotinus* south of the Pyrenees and describe their evolutionary relationships (Fig 1A and 1B). Two of them grouped within the recently described cluster of EBLV-1a sequences from Southern France [6], extending this group now to Northern Spain. The EBLV-1a subtype is otherwise typically distributed through Northern Europe, along an east-west axis from The Netherlands to Ukraine [5]. The shallow genetic differentiation (Figs 1B and 2A) found between the strains on both slopes of the Pyrenees of this Southern group of EBLV-1a suggests a recent geographical expansion of this subtype across southern France, with very recent arrival to the Iberian Peninsula. On the other hand, one of the new EBLV-1a strains was found only 24 km away from the nearest Iberian EBLV-1b strain (Fig 1A), with no significant geographical barriers between them. This supports a hypothesis of current southwards expansion of EBLV-1a, as was proposed for Western Europe by Vázquez-Morón et al. [7]. The clear star-like structure observed for EBLV-1a in the haplotype network provides additional support for this hypothesis (Fig 2A).

The remaining seven new EBLV-1 sequences detected in *E*. *serotinus* cluster together with the Iberian clade that had been exclusively found in *E*. *isabellinus* in the Southern half of the Iberian Peninsula [7] (Fig 1B). This Iberian clade was considered to be differentiated from both EBLV-1a and EBLV-1b [7]. However, our new phylogenetic analyses show that this Iberian lineage shared by the two species of *Eptesicus* is actually part of the EBLV-1b subtype. This fact points again to a more predominant role of geographical factors than the host species in determining EBLV-1’s phylogenetic structure in Iberia. Both *Eptesicus* species are phylogenetically close and show some overlap in their geographical distribution in Iberia [13]. Interestingly, phylogenetic relatedness and geographic contact, were considered the two main conditions for interspecific transmission of rabies among bats in America [14]. The haplotype network (Fig 2B) shows a much more complex structure and higher haplotype diversity for EBLV-1b than for EBLV-1a, suggesting a much longer evolutionary history. The analyses also suggest that Iberian EBLV-1b has evolved isolated from the rest of European EBLV-1b, from a still unknown common ancestor, although more geographic coverage is still necessary to fully reconstruct this event.

Our coalescence based analysis (S3 Fig) suggests that the Most Recent Common Ancestor (tMRCA) for the entire EBLV-1 lineage currently circulating in *Eptesicus* bats dates to 175 years ago ([47; 260] HDP). This value is remarkably similar (just five years older) to the date previously proposed [12], but with narrower 95% HDP intervals. The tMRCA estimated for the whole EBLV-1a lineage was approximately 70 years ([59; 158] HDP), while the Iberian EBLV-1a strains could have emerged less than 15 years ago, ([4; 13] HDP) giving additional support to the hypothesis of a recent southward expansion from France across the Pyrenees.

On the other hand, the tMRCA for the subtype EBLV-1b is estimated be around 110 years ago, ([53; 187] HDP). This result suggests a longer evolutionary history than EBLV-1a in agreement with the Network analysis. The Iberian EBLV-1b strains would have spawned approximately 55 years ago, ([31; 82] HDP). Nevertheless, we suggest caution on this dating since some uncertainty for the marginal posterior distribution of all the tMRCA was noticed, which will, most probably, be solved when additional cases of Iberian EBLV-1 infection are available.

**Fig 3:**
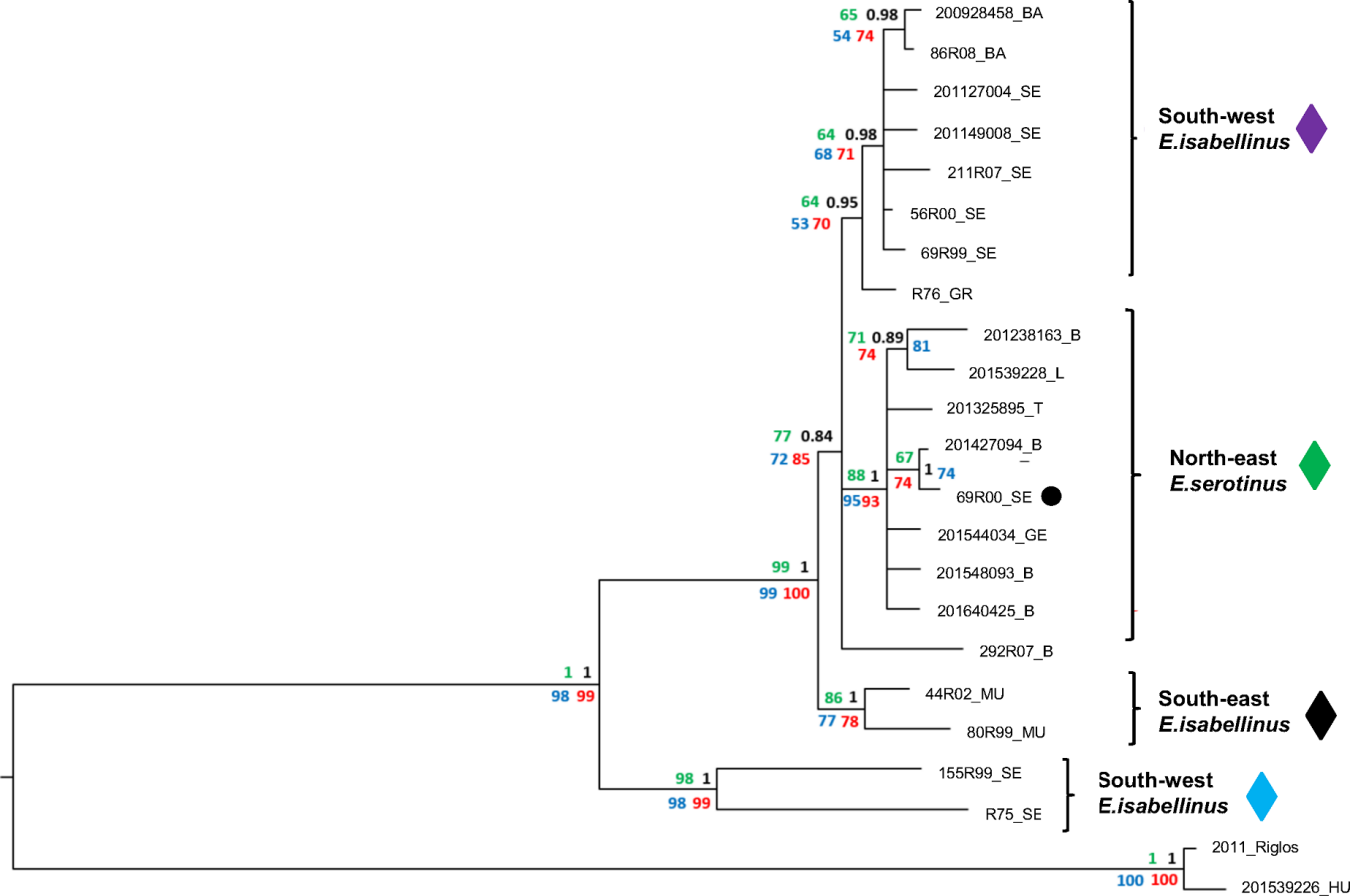
Iberian EBLV-1b midpoint rooted bayesian phylogenetic reconstruction based on 1,809 bp sequences of the concatenated fragments of nucleoprotein, phosphoprotein and glycoprotein-polymerase’s intergenic region. Tree nodes show posterior probability (black) and Maximum-Parsimony (red), Neighbor-Joining (blue) and Maximum-Likelihood (green) bootstrap support values. Two Iberian EBLV-1a sequences (2011_Riglos, 201539226_HU) have been included as outgroup. Color code of the diamonds associated to the main groups is the same than in Fig 1A. Two Iberian EBLV-1a sequences (2011_Riglos, 201539226_HU) are included as outgroup. The sequence 69R00_SE, marked with a black dot within the north-east-*E*. *serotinus* group, was obtained from an *E*. *isabellinus* from the south-west (see text).

In addition to the nucleoprotein gene, the two hypervariable regions studied enlighten the internal structure within the Iberian EBLV-1b cluster. The strains associated with *E*. *serotinus* seem particularly related to those associated with *E*. *isabellinus* from the South West (Fig 3). In fact, the presence of a strain (69R00_SE) from Seville (South West) in the new group of viruses associated with *E*. *serotinus* (North East) points to an inter-specific transmission of EBLV-1 (Fig 1A and Fig 3) and suggests that this particular Iberian lineage has extended northwards from *E*. *isabellinus* to *E*. *serotinus*. In fact, the Network shows the Northern Iberian EBLV-1b strains associated with *E*. *serotinus* branching off from a common Southern haplotype (in an expansion star-like shape), providing additional support for this hypothesis (Fig 2B). On the other hand, the Iberian South West seems to concentrate the highest variability within the Iberian EBLV-1b lineage (Fig 1A), including a sub-lineage represented by two sequences (155R99, R75) which occupied the most basal position and have never been detected after 1999 (Fig 3). An east-west discrimination of two groups of viruses in the South is in agreement with the east-west population subdivision described for the host bat *E*. *isabellinus* [9]. Interestingly, this study suggests a close genetic relatedness between South Western Iberian and North-African populations for *E*. *isabellinus* [9]. This genetic pattern strongly supports the possibility of the presence of EBLV-1 in North Africa from where it has not been reported yet.

In conclusion, the increasing involvement of wild life rescue centers in bat rabies surveillance has allowed us to detect the first cases in Iberian *E*. *serotinus* and we hope it will allow us to increase the overall number of Iberian strains in the upcoming years. Unfortunately, active screening of healthy bats captured in colonies by testing oral fluids and blood does not usually provide enough genomic material to perform this kind of study [15]. The use of highly variable genomic regions (Fig 3, S3A and S3B Fig) has clearly improved the resolution of the virus evolutionary phylogenetic reconstructions [16], and has provided a better understanding of the phylogenetic structure of the Iberian EBLV-1 populations, pointing to evolutionary patterns at a continental level that deserve further research.

## Supporting information

S1 Table All EBLV-1 strains used and their characteristics, including accession numbers of all sequences. (PDF)

S1 Fig Iberian EBLV-1b Bayesian phylogenetic reconstruction based on 686 bp sequences of phosphoprotein after sampling 10,000,000 tree generations. Tree nodes show posterior probability. Two Iberian EBLV-1a sequences (2011_Riglos, 201539226_HU) have been included as outgroup.
(TIF)

S2 Fig Iberian EBLV-1b Bayesian phylogenetic reconstruction based on 763bp of 5' terminal sequences of glycoprotein-polymerase’s intergenic region after sampling 10,000,000 tree generations. Tree nodes show posterior probability. Two Iberian EBLV-1a sequences (2011_Riglos, 201539226_HU) have been included as outgroup.
(TIF)

S3 Fig Maximum clade credibility (MCC) phylogeny for all EBLV-1 nucleoprotein sequences. Blue node bars represent 95% highest posterior density (95% HPD) as Bayesian credible interval. Posterior values are shown as color-coded node dots, showing red as the highest posterior value.
(TIF)

## References

[1] Arechiga Ceballos N, Vazquez Moron S, Berciano JM, Nicolas O, Aznar Lopez C, Juste J, et al Novel lyssavirus in bat, Spain. Emerg Infect Dis 2013 5;19(5):793–795. doi: 10.3201/eid1905.121071 23648051 10.3201/eid1905.121071 PMC3647500

[2] Gunawardena PS, Marston DA, Ellis RJ, Wise EL, Karawita AC, Breed AC, et al Lyssavirus in Indian Flying Foxes, Sri Lanka. Emerg Infect Dis. 2016;22(8):1456–1459. doi: 10.3201/eid2208.151986 27434858 10.3201/eid2208.151986 PMC4982157

[3] Botvinkin AD, Poleschuk EM, Kuzmin IV, Borisova TI, Gazaryan SV, Yager P, et al Novel lyssaviruses isolated from bats in Russia. Emerg Infect Dis 2003 12;9(12):16231625. 10.3201/eid0912.030374 PMC3034350 14720408

[4] Schatz J, Fooks AR, McElhinney L, Horton D, Echevarria J, Vazquez-Moron S, et al Bat rabies surveillance in Europe. Zoonoses Public Health 2013 2;60(1):22–34. doi: 10.1111/zph.12002 22963584 10.1111/zph.12002

[5] McElhinney LM, Marston DA, Leech S, Freuling CM, van der Poel WH, Echevarria J, et al Molecular epidemiology of bat lyssaviruses in Europe. Zoonoses Public Health 2013 2;60(1):35–45. doi: 10.1111/zph.12003 22937876 10.1111/zph.12003

[6] Picard-Meyer E, Robardet E, Arthur L, Larcher G, Harbusch C, Servat A, et al Bat rabies in France: a 24-year retrospective epidemiological study. PLoS One 2014 6 3;9(6):e98622 doi: 10.1371/journal.pone.0098622 24892287 10.1371/journal.pone.0098622 PMC4044004

[7] Vazquez-Moron S, Juste J, Ibanez C, Berciano JM, Echevarria JE. Phylogeny of European bat Lyssavirus 1 in Eptesicus isabellinus bats, Spain. Emerg Infect Dis 2011 3;17(3):520–523. doi: 10.3201/eid1703.100894 21392449 10.3201/eid1703100894 PMC3166003

[8] Ibáñez C, García-Mudarra O, Ruedi S,B, Juste J. The Iberian Contribution to Cryptic Diversity in European Bats. Proc Biol Sci 2006 9 7;8(2):277–297.

[9] Juste J, Bilgin R, Munoz J, Ibanez C. Mitochondrial DNA signatures at different spatial scales: from the effects of the Straits of Gibraltar to population structure in the meridional serotine bat (Eptesicus isabellinus). Heredity (Edinb) 2009 8;103(2):178–187. 19401715 10.1038/hdy.2009.47

[10] Vazquez-Moron S, Avellon A, Echevarria JE. RT-PCR for detection of all seven genotypes of Lyssavirus genus. J Virol Methods 2006 8;135(2):281–287. doi: 10.1016/j.jviromet.2006.03.008 16713633 10.1016/j.jviromet.2006.03.008

[11] Drummond AJ, Suchard MA, Xie D, Rambaut A. Bayesian phylogenetics with BEAUti and the BEAST 1.7. Mol Biol Evol 2012 8;29(8):1969–1973. doi: 10.1093/molbev/mss075 22367748 10.1093/molbev/mss075 PMC3408070

[12] Hughes GJ. A reassessment of the emergence time of European bat lyssavirus type 1. Infect Genet Evol 2008 12;8(6):820–824. doi: 10.1016/j.meegid.2008.08.003 18773973 10.1016/j.meegid.2008.08.003

[13] Santos H, Juste J, Ibáñez C, Palmeirim JM, Godinho R, Amorim F, et al Influences of ecology and biogeography on shaping the distributions of cryptic species: three bat tales in Iberia. Biol J Linn Soc 2014;112(1):150–162.

[14] Streicker DG, Turmelle AS, Vonhof MJ, Kuzmin IV, McCracken GF, Rupprecht CE. Host phylogeny constrains cross-species emergence and establishment of rabies virus in bats. Science 2010 8 6;329(5992):676–679. doi: 10.1126/science.1188836 20689015 10.1126/science.1188836

[15] Vazquez-Moron S, Juste J, Ibanez C, Aznar C, Ruiz-Villamor E, Echevarria JE. Asymptomatic rhabdovirus infection in meridional serotine bats (Eptesicus isabellinus) from Spain. Dev Biol (Basel) 2008;131:311–316. 18634493

[16] Gadagkar SR, Rosenberg MS, Kumar S. Inferring species phylogenies from multiple genes: concatenated sequence tree versus consensus gene tree. J Exp Zool B Mol Dev Evol 2005 1 15;304(1):64–74. doi: 10.1002/jez.b.21026 15593277 10.1002/jez.b.21026

