# Supplementary material for: First cases of European bat lyssavirus type 1 in Iberian serotine bats: Implications for the molecular epidemiology of bat rabies in Europe
Source: PLoS Negl Trop Dis. 2018 Apr 23;12(4):e0006290. doi: 10.1371/journal.pntd.0006290 (PMC5933805; doi:10.1371/journal.pntd.0006290)
Supplement: S1 Table — (PDF) [file pntd.0006290.s001.pdf]

S1 Table: All EBLV-1 strains used and their characteristics.

Identification	Subtype	Year	Country	N-400	P	G-L
0001FR	EBLV-1b	2000	France; Premilhat	AY863396	-	-
126235FR	EBLV-1b	2005	France; Unknown	KJ210399	-	-
128827FR	EBLV-1b	2009	France; Centre France	KC567814	-	-
129290FR	EBLV-1b	2011	France; Centre France	KJ210400	-	-
3400FR	EBLV-1b	2001	France; Vallon en Sully	AY863400	-	-
9600FR	EBLV-1b	1995	France; Bourges	AY863394	-	-
3701FR	EBLV-1b	2001	France; Unknown	AY245837	-	-
127051FR	EBLV-1b	2006	France; Centre France	KC567815	-	-
129246FR	EBLV-1b	2011	France; Centre France	KJ210398	-	-
3202FR	EBLV-1b	2002	France; Unknown	AY245832	-	-
0101FR	EBLV-1b	2001	France; Unknown	AY863401	-	-
9700FR	EBLV-1b	2000	France; Unknown	AY863397	-	-
124345FR	EBLV-1b	2004	France; Northeast France	KC567812	-	-
126669FR	EBLV-1b	2005	France; Northeast France	KC567813	-	-
129123FR	EBLV-1b	2010	France; Unknown	KJ210397	-	-
9800FR	EBLV-1b	2000	France; Unknown	AY863398	-	-
129666FR	EBLV-1b	2012	France; Northeast France	KJ210394	-	-
9495FR	EBLV-1b	1995	France; Unknown	AY863394	-	-
129865FR	EBLV-1b	2012	France; Northeast France	KJ210396	-	-
4498FR	EBLV-1b	1998	France; Unknown	AY245844	-	-
9595FR	EBLV-1b	1995	France; Unknown	AY863395	-	-
78983FR	EBLV-1b	1989	France; Brainville	KJ210395	-	-
9389FR	EBLV-1b	1989	France; Unknown	AY863393	-	-
0201FR	EBLV-1b	2001	France; Unknown	AY863402	-	-
128636FR	EBLV-1b	2009	France; Northeast France	KC567817	-	-
128665FR	EBLV-1b	2009	France; Unknown	KC567818	-	-
3300FR	EBLV-1b	2000	France; Unknown	AY245833	-	-
4197FR	EBLV-1b	1997	France; Unknown	AY245841	-	-
4201FR	EBLV-1b	2001	France; Unknown	AY245842	-	-
9900FR	EBLV-1b	2000	France; Unknown	AY863399	-	-
129087FR	EBLV-1b	2010	France; Northeast France	KC567816	-	-
129394FR	EBLV-1b	2011	France; Northeast France	KJ210402	-	-
129409FR	EBLV-1b	2011	France; Northeast France	KJ210403	-	-

129290FR	EBLV-1b	2011	France; Centre France	KJ210400	-	-
129428FR	EBLV-1b	2011	France; Northeast France	KF186270	-	-
8918FR	EBLV-1b	1989	France; Unknown	GU992305	-	-
RV22FR	EBLV-1b	-	France; Unknown	DQ522894	-	-
120116FR	EBLV-1a	2010	France; Northeast France	KJ210401	-	-
123801FR	EBLV-1a	2003	France; Northeast France	KJ210405	-	-
124193FR	EBLV-1a	2004	France; Northeast France	KJ210404	-	-
128210FR	EBLV-1a	2008	France; Northeast France	KC567810	-	-
129051FR	EBLV-1a	2010	France; Northeast France	KC567809	-	-
810FR	EBLV-1a	2003	France; Angers	AY863381	-	-
129055FR	EBLV-1a	2010	France; Northeast France	KC567808	-	-
129944FR	EBLV-1a	2012	France; Unknown	KC567811	-	-
124352FR	EBLV-1a	-	France; Unknown	AF124352	-	-
124353FR	EBLV-1a	-	France; Unknown	AF124353	-	-
124354FR	EBLV-1a	-	France; Unknown	AF124354	-	-
28228GR	EBLV-1b	2012	Germany; Chemnitz	KF826121	-	-
11647GR	EBLV-1a	2005	Germany; Brandenburg	KF826097	-	-
18814GR	EBLV-1a	2007	Germany; Magdeburg	KF826102	-	-
23544GR	EBLV-1a	2009	Germany; Halle-Saale	KF826109	-	-
25006GR	EBLV-1a	2003	Germany; Langenhagen	KF826117	-	-
27904GR	EBLV-1a	2011	Germany; Halle-Saale	KF826120	-	-
20170GR	EBLV-1a	2008	Germany; Halle-Saale	KF826105	-	-
32054GR	EBLV-1a	2012	Germany; Berlin	KF826126	-	-
5589GR	EBLV-1a	1989	Germany; Braunschweig	AY863355	-	-
24832GR	EBLV-1a	-	Germany; Berlin	KF826116	-	-
31448GR	EBLV-1a	1993	Germany; Unknown	KF042302	-	-
915GR	EBLV-1a	1997	Germany; Emden	KF826139	-	-
15730GR	EBLV-1a	2005	Germany; Lubbenan	KF826096	-	-
16902GR	EBLV-1a	-	Germany; Aurich	KF826098	-	-
16908GR	EBLV-1a	2004	Germany; Stedesdorf	KF826099	-	-
18720GR	EBLV-1a	2002	Germany; Thedinghausen	KF826100	-	-
19926GR	EBLV-1a	2008	Germany; Nordhorn	KF826104	-	-

30575GR	EBLV-1a	2010	Germany; Hameln	KF826122	-	-
5248GR	EBLV-1a	-	Germany; Bremen	KF826131	-	-
905GR	EBLV-1a	-	Germany; Zippelsforde	KF826138	-	-
934GR	EBLV-1a	1997	Germany; Osnabruck	KF826142	-	-
5786GR	EBLV-1a	1896	Germany; Nienburg	AY863357	-	-
933GR	EBLV-1a	1993	Germany; Oldendorf	KF826141	-	-
21836GR	EBLV-1a	2010	Germany; Bergen	KF826101	-	-
24525GR	EBLV-1a	2010	Germany; Moisburg	KF826110	-	-
24529GR	EBLV-1a	-	Germany; Verden	KF826111	-	-
24610GR	EBLV-1a	2000	Germany; Lower Saxony	KF826112	-	-
5185GR	EBLV-1a	2000	Germany; Hitzhausen	KF826129	-	-
5254GR	EBLV-1a	-	Germany; Osterode	KF826133	-	-
24831GR	EBLV-1a	-	Germany; Berlin	KF826114	-	-
32062GR	EBLV-1a	2012	Germany; Berlin	KF826127	-	-
25495GR	EBLV-1a	2010	Germany; Hamburg	KF826118	-	-
31054GR	EBLV-1a	2010	Germany; Bad Segeberg	KF826123	-	-
24610GR	EBLV-1a	2000	Germany; Lower Saxony	KF826112	-	-
5185GR	EBLV-1a	2000	Germany; Hitzhausen	KF826129	-	-
5254GR	EBLV-1a	-	Germany; Osterode	KF826133	-	-
24831GR	EBLV-1a	-	Germany; Berlin	KF826114	-	-
32062GR	EBLV-1a	2012	Germany; Berlin	KF826127	-	-
25495GR	EBLV-1a	2010	Germany; Hamburg	KF826118	-	-
31054GR	EBLV-1a	2010	Germany; Bad Segeberg	KF826123	-	-
31177GR	EBLV-1a	2011	Germany; North Rhine	KF826124	-	-
31178GR	EBLV-1a	2011	Germany; Hamminkeln	KF826125	-	-
4644GR	EBLV-1a	1997	Germany; Plate	KF826128	-	-
4868GR	EBLV-1a	1968	Germany; Hamburg	AY863348	-	-
4985GR	EBLV-1a	1985	Germany; Rostock	AY863349	-	-
8268GR	EBLV-1a	1968	Germany; Unknown	AY062082	-	-
9394GR	EBLV-1a	1968	Germany; Hamburg	U89466	-	-
5182GR	EBLV-1a	1982	Germany; Bremerhaven	AY863351	-	-
5226GR	EBLV-1a	1996	Germany; Lingen	KF826130	-	-

5250GR	EBLV-1a	1994	Germany; Hannover	KF826132	-	-
5286GR	EBLV-1a	1986	Germany; Stade,	AY863352	-	-
5300GR	EBLV-1a	1999	Germany; Emden	KF826134	-	-
5304GR	EBLV-1a	1999	Germany; Emden	KF826135	-	-
5387GR	EBLV-1a	1987	Germany; Bremen	AY863353	-	-
5488GR	EBLV-1a	1988	Germany; Neumunster	AY863354	-	-
5070GR	EBLV-1a	1970	Germany; Stade	AY863350	-	-
5690GR	EBLV-1a	1990	Germany; Walsrode	AY863356	-	-
5890GR	EBLV-1a	1990	Germany; Lubeck	AY863358	-	-
6189GR	EBLV-1a	1989	Germany; Bad Iberg	U89461	-	-
6490GR	EBLV-1a	1990	Germany; Ratzeburg	U89464	-	-
7467GR	EBLV-1a	2000	Germany; Emden	KF826136	-	-
7471GR	EBLV-1a	2000	Germany; Emden	KF826137	-	-
83XXGR	EBLV-1a	-	Germany; Unknown	AY062083	-	-
959GR	EBLV-1a	1988	Germany; Aurich	KF826143	-	-
992GR	EBLV-1a	1992	Germany; Moordof	KF826146	-	-
998GR	EBLV-1a	1997	Germany; Emden	KF826147	-	-
B24	EBLV-1a	1986	Germany; Unknown	KF831560	-	-
BATSTADE	EBLV-1a	1970	Germany; Unknown	KF831550	-	-
932GR	EBLV-1a	1991	Germany; Breddenburg	KF826140	-	-
976GR	EBLV-1a	1992	Germany; Marienhafe	KF826144	-	-
989GR	EBLV-1a	-	Germany; Aurich	KF826145	-	-
DR707LX	EBLV-1b	2013	Luxemburg; Differdange	KF186269	-	-
4992HO	EBLV-1b	1992	The Netherlands; Unknown	U89449	-	-
8997HO	EBLV-1b	1997	The Netherlands; Unknown	AY863389	-	-
8392HO	EBLV-1b	1992	The Netherlands; Unknown	AY863383	-	-
8493HO	EBLV-1b	1993	The Netherlands; Unknown	AY863384	-	-
8593HO	EBLV-1b	1993	The Netherlands; Unknown	AY863385	-	-
8692HO	EBLV-1b	1992	The Netherlands; Unknown	AY863386	-	-
8792HO	EBLV-1b	1992	The Netherlands; Unknown	AY863387	-	-
8899HO	EBLV-1b	1999	The Netherlands; Unknown	AY863388	-	-
5493HO	EBLV-1a	1993	The Netherlands; Unknown	U89454	-	-

6189HO	EBLV-1a	1989	The Netherlands; Rolde	AY863361	-	-
6500HO	EBLV-1a	2000	The Netherlands; Leiden	AY863365	-	-
6699HO	EBLV-1a	1999	The Netherlands; Woerden	AY863366	-	-
7387DK	EBLV-1a	1987	Denmark; Horsens	AY863373	-	-
6092HO	EBLV-1a	1992	The Netherlands; Valthermond	AY863360	-	-
6287HO	EBLV-1a	1987	The Netherlands; Bellingwolde	AY863362	-	-
6389HO	EBLV-1a	1989	The Netherlands; Jubbega	AY863363	-	-
6400HO	EBLV-1a	2000	The Netherlands; Soest	AY863364	-	-
6885HO	EBLV-1a	1985	The Netherlands; Unknown	AY893368	-	-
7487DK	EBLV-1a	1987	Denmark; Christiansfeld	AY863374	-	-
7595DK	EBLV-1a	1995	Denmark; Arden,	AY863375	-	-
7687HO	EBLV-1a	1987	The Netherlands; Joure	U89476	-	-
RV31HO	EBLV-1a	1998	The Netherlands; Unknown	KF831551	-	-
7697DK	EBLV-1a	1997	Denmark; Jerslev	AY863376	-	-
8002DK	EBLV-1a	2002	Denmark; Janderup,	AY863380	-	-
85XXDK	EBLV-1a	-	Denmark; Unknown	AY062085	-	-
7799DK	EBLV-1a	1999	Denmark; Unknown	AY86337	-	-
7899DK	EBLV-1a	1999	Denmark; Nr.Nebel	AY863378	-	-
7900DK	EBLV-1a	2000	Denmark; Rødding,	AY863379	-	-
84XXDK	EBLV-1a	-	Denmark; Unknown	AY062084	-	-
BATDANMARK	EBLV-1a	1986	Denmark; Unknown	KF831549	-	-
RV20DK	EBLV-1a	-	Denmark; Unknown	AY062085	-	-
Hun_bat_1999_1HU	EBLV-1a	1999	Hungary; Unknown	KC692770	-	-
Hun_bat_2009_1HU	EBLV-1a	2009	Hungary; Unknown	KC692771	-	-
Hun_bat_2010_1HU	EBLV-1a	2010	Hungary; Unknown	KC692772	-	-
Hun_bat_2011_1HU	EBLV-1a	2011	Hungary; Unknown	KC692773	-	-
Hun_bat_2011_2HU	EBLV-1a	2011	Hungary; Unknown	KC692774	-	-
Hun_bat_2012_1HU	EBLV-1a	2012	Hungary; Unknown	KC692775	-	-
5594PO	EBLV-1a	1994	Poland; Nietoperz	U89455	-	-

6985PO	EBLV-1a	1985	Poland; Gdansk	AY863369	-	-
7185RU	EBLV-1a	1985	Rusia; Belgorod	AY863371	-	-
7287UC	EBLV-1a	1987	Ukraine; Volyn	AY863372	-	-
87XXPO	EBLV-1a	-	Poland; Unknown	AY062087	-	-
155R99SP	EBLV-1b	1999	Spain; Seville	DQ222419	MG460576	MG460569
R75SP	EBLV-1b	1989	Spain; Seville	DQ222421	KY087917	KY087936
200928458SP	EBLV-1b	2009	Spain; Badajoz	HM212663	KY087900	KY087920
201427094SP	EBLV-1b	2014	Spain; Barcelona	KY087943	KY087906	KY087928
201539228SP	EBLV-1b	2015	Spain; Huesca	KY087942	KY087907	KY087925
201544034SP	EBLV-1b	2015	Spain; Gerona	KY087941	KY087908	KY087926
201548093SP	EBLV-1b	2015	Spain; Barcelona	KY087940	KY087909	KY087927
211R07SP	EBLV-1b	2007	Spain; Seville	HM212662	KY087910	KY087929
292R07SP	EBLV-1b	2007	Spain; Granada	HM212661	KY087911	KY087930
44R02SP	EBLV-1b	2002	Spain; Murcia	DQ222425	KY087912	KY087931
80R99SPSP	EBLV-1b	1999	Spain; Murcia	DQ222424	KY087915	KY087934
56R00SP	EBLV-1b	2000	Spain; Seville	KY087945	MG460575	MG460568
69R00SP	EBLV-1b	2000	Spain; Seville	DQ222420	KY087913	KY087932
69R99SP	EBLV-1b	1999	Spain; Seville	DQ222423	KY087914	KY087933
86R08SP	EBLV-1b	2008	Spain; Badajoz	HM212664	KY087916	KY087935
R76SP	EBLV-1b	1987	Spain; Granada	DQ222422	KY087918	KY087937
201539226SP	EBLV-1a	2015	Spain; LLeida	KY087939	KY087899	MG460571
2011riglosSP	EBLV-1a	2011	Spain; Huesca	KY087938	MG460578	KY087919
201238163SP	EBLV-1b	2012	Spain; Barcelona	MG460572	KY087903	KY087923
201325895SP	EBLV-1b	2013	Spain, Tarragona	MG460573	KY087904	KY087924
201640425SP	EBLV-1b	2016	Spain Barcelona	MG460574	MG460577	MG460570
201127004SP	EBLV-1b	2011	Spain; Seville	MG765418	KY087901	KY087921
201149008SP	EBLV-1b	2011	Spain; Seville	MG765419	KY087902	KY087922

Table footnotes: - Missing data
