# Supplementary figures and images for: First cases of European bat lyssavirus type 1 in Iberian serotine bats: Implications for the molecular epidemiology of bat rabies in Europe
Source: PLoS Negl Trop Dis. 2018 Apr 23;12(4):e0006290. doi: 10.1371/journal.pntd.0006290 (PMC5933805; doi:10.1371/journal.pntd.0006290)

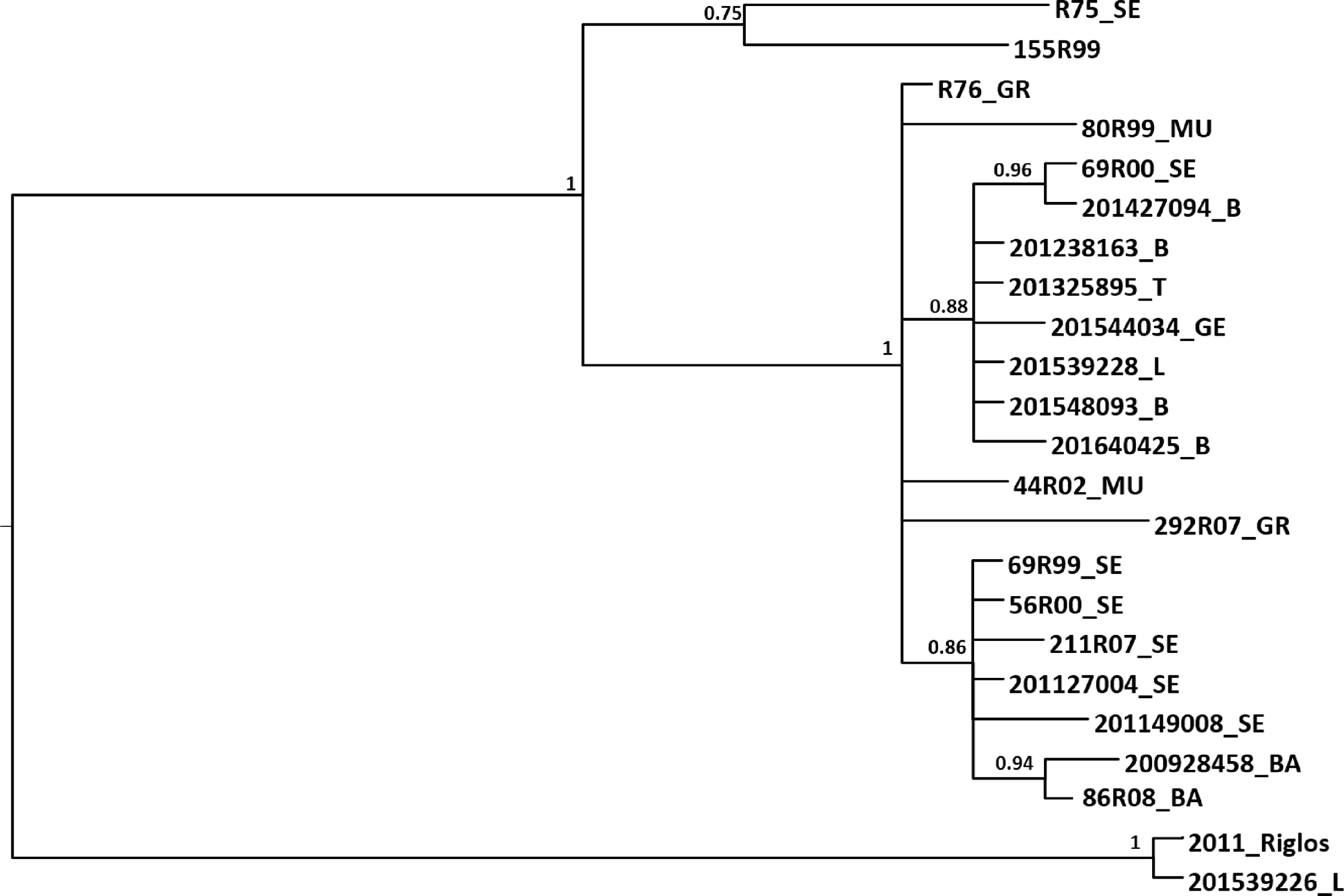

Supplement: S1 Fig — Tree nodes show posterior probability. Two Iberian EBLV-1a sequences (2011_Riglos, 201539226_HU) have been included as outgroup. (TIF) [file pntd.0006290.s002.tif]

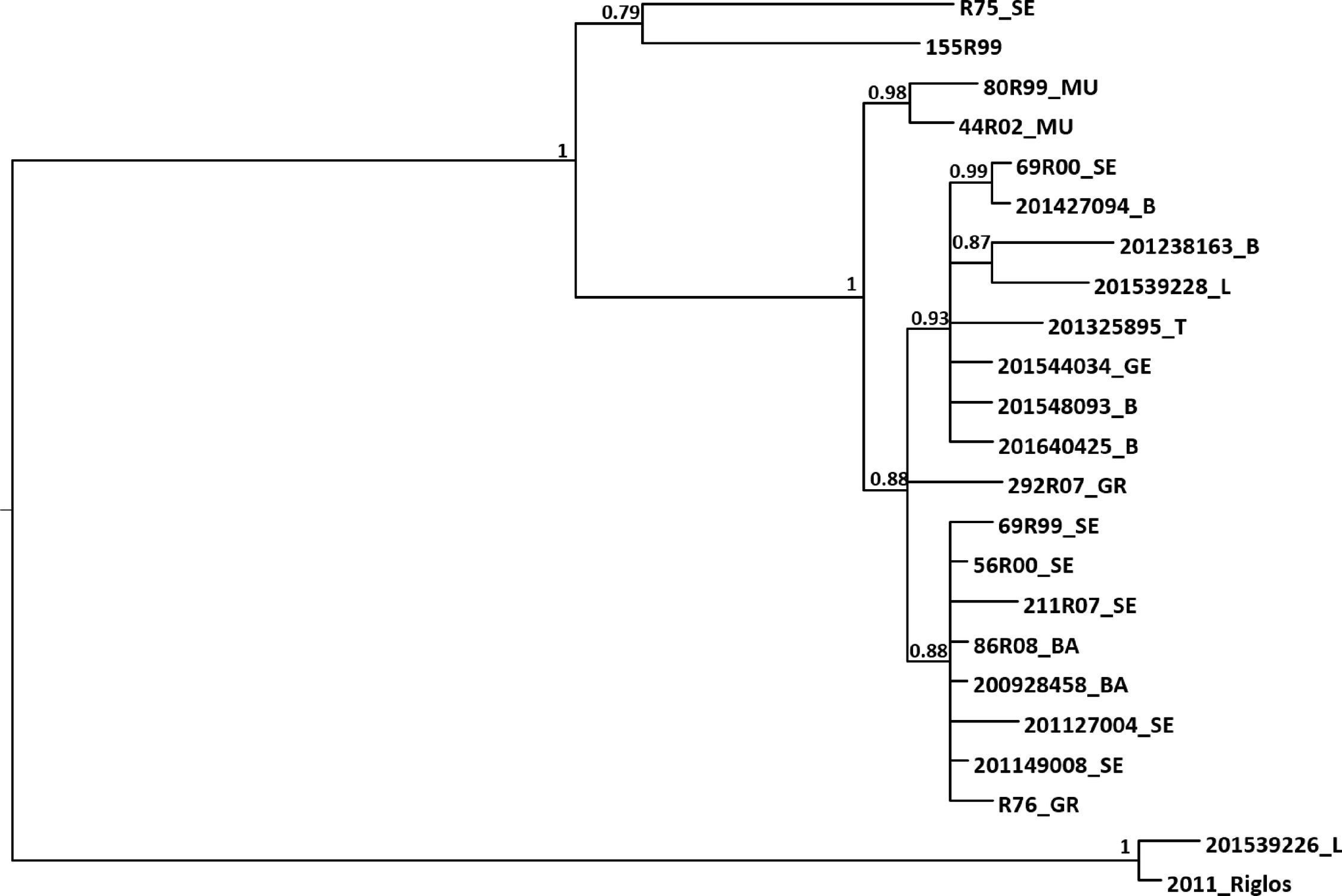

Supplement: S2 Fig — Tree nodes show posterior probability. Two Iberian EBLV-1a sequences (2011_Riglos, 201539226_HU) have been included as outgroup. (TIF) [file pntd.0006290.s003.tif]

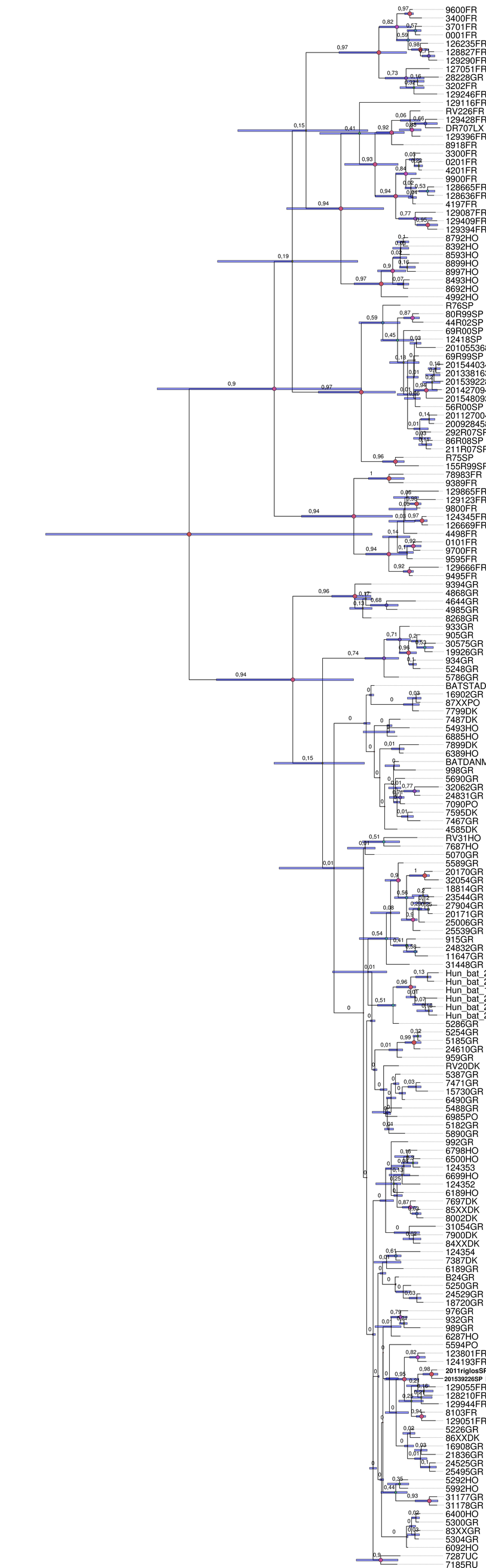

Supplement: S3 Fig — Blue node bars represent 95% highest posterior density (95% HPD) as Bayesian credible interval. Posterior values are shown as color-coded node dots, showing red as the highest posterior value. (TIF) [file pntd.0006290.s004.tif]
